# Low-dose nivolumab in advanced hepatocellular carcinoma

**DOI:** 10.1186/s12885-022-10271-6

**Published:** 2022-11-08

**Authors:** Yen-Hao Chen, Chih-Chi Wang, Yen-Yang Chen, Jing-Houng Wang, Chao-Hung Hung, Yuan-Hung Kuo

**Affiliations:** 1 Division of Hematology-Oncology, Department of Internal Medicine, Kaohsiung Chang Gung Memorial Hospital and Chang Gung University College of Medicine, No.123, Dapi Rd., Niaosong Dist, Kaohsiung City, 833 Taiwan; 2 School of Medicine, College of Medicine, Chang Gung University, Taoyuan, 333 Taiwan; 3 School of Medicine, Chung Shan Medical University, Taichung, 402 Taiwan; 4 Department of nursing, School of nursing, Fooyin University, Kaohsiung, 831 Taiwan; 5 Division of General Surgery, Department of Surgery, Kaohsiung Chang Gung Memorial Hospital and Chang Gung University College of Medicine, Kaohsiung, 833 Taiwan; 6 Division of Hepatogastroenterology, Department of Internal Medicine, Kaohsiung Chang Gung Memorial Hospital and Chang Gung University College of Medicine, Kaohsiung, 833 Taiwan

**Keywords:** Nivolumab, Low dose, Hepatocellular carcinoma, Immunotherapy

## Abstract

**Background:**

The approved dose of nivolumab is 3 mg/kg or a flat dose of 240 mg for indications. There is no dose-response relationship for nivolumab; therefore, a low-dose regimen may be an option to reduce financial toxicity. This study was designed to investigate the efficacy and safety of low-dose nivolumab in the management of hepatocellular carcinoma (HCC).

**Methods:**

We retrospectively reviewed patients with HCC who received 20 or 100 mg of nivolumab intravenously every 2 weeks. The objective response rate was determined in accordance with the Response Evaluation Criteria in Solid Tumors criteria version 1.1. The Cox regression model and Kaplan–Meier method were used to analyze hazard factors, progression-free survival (PFS), and overall survival (OS). Adverse events (AEs) were assessed according to the National Cancer Institute Common Terminology Criteria for Adverse Events version 5.0.

**Results:**

In total, 78 patients were enrolled, including 49 with hepatitis B virus (HBV) and 23 with hepatitis C virus (HCV). All patients were staged as Barcelona Clinic Liver Cancer stage C, and 20 patients were classified as having Child–Pugh classification B (7). Nivolumab 20 mg was an independent prognostic factor for better PFS, and albumin-bilirubin grade 1 was the independent prognostic factor for superior OS in the multivariate analyses. Patients with better HBV (HBV DNA < 500 IU/ml) and HCV (HCV RNA undetectable) controls had superior OS. All AEs were grade 1–2 in severity, and all patients tolerated nivolumab without treatment interruption or dose adjustment. Additionally, 31 patients underwent subsequent therapy after nivolumab treatment.

**Conclusion:**

Low-dose nivolumab may be effective with manageable toxicity and can be an alternative option to reduce financial toxicity in patients with advanced HCC who cannot afford the high cost of immune checkpoint inhibitors in real-world practice.

## Introduction

Hepatocellular carcinoma (HCC) is the most common primary liver cancer with increasing incidence and cancer-related death; in several Asian countries including Taiwan, it also is the leading cause of mortality [1]. Multi-kinase inhibitors, such as sorafenib, have been approved for systemic therapy in patients with advanced HCC for > 10 years; subsequently, lenvatinib, regorafenib, and cabozantinib have also been proved for HCC treatment by randomized phase II or III controlled trials [2–7]. Recently, immunotherapy has developed as a novel approach for the management of cancer, and immune checkpoint inhibitors (ICIs), which target programmed death 1 (PD-1) or programmed death-ligand 1, have revolutionized the strategy of oncology and become the standard treatment in several cancers, such as melanoma or non-small cell lung cancer (NSCLC) [8, 9]. In HCC, atezolizumab plus bevacizumab has shown superior results in terms of objective response rate (ORR), progression-free survival (PFS) and overall survival (OS) compared to those of sorafenib in patients with advanced HCC and become the a new first-line systemic treatment of HCC [10]. In addition to the approval of atezolizumab plus bevacizumab, other ICIs such as pembrolizumab or nivolumab, have been approved for HCC treatment after sorafenib progression based on the efficacy and OS improvement [11, 12]. Therefore, successful cancer immunotherapy has created a break-through in the field of HCC treatment.

Nivolumab, an anti-PD-1 monoclonal antibody, improves survival in several cancer types. Currently, the Food and Drug Administration (FDA) has granted accelerated approval for the combination of nivolumab plus ipilimumab (an anti-cytotoxic T-lymphocyte-associated protein 4 antibody) for second-line systemic treatment in patients with advanced HCC; however, nivolumab alone has been proven to treat advanced HCC in Taiwan based on early phase trials [11, 13, 14]. Reimbursement of expensive drugs, such as ICIs, is relatively difficult in resource-constrained areas; therefore, nivolumab is reimbursed only in a few Asian countries. Therefore, ICI use in clinical practice is hampered by the extremely high cost, resulting in unaffordability to many cancer patients and poor survival outcomes. Therefore, low-dose ICIs may be a viable option for the management of cancer. Growing evidence has demonstrated the efficacy of low-dose nivolumab in some cancer types, such as NSCLC, recall cell carcinoma (RCC), and Hodgkin lymphoma [15–17]. The major rationale is that no correlation between dose and response has been observed for anti-PD-1 ICIs, whether nivolumab or pembrolizumab. In a phase 1 study of nivolumab in HCC, the doses were prescribed from 0.1 to 10 mg/kg every 2 weeks (Q2W) [11]. Subsequently, the FDA approved nivolumab at a flat dose of 240 mg Q2W for all approved indications regardless of body weight based on the comparability of the pharmacokinetic exposure, safety, and efficacy [18]. Nevertheless, the efficacy of nivolumab at lower doses has been mentioned in patients with RCC in early phase studies [19, 20].

In Taiwan, most patients do not have adequate reimbursement plans or national income to afford ICIs. A low-dose regimen may be an alternative option to reduce the financial toxicity incurred by ICIs. The efficacy and safety of low-dose nivolumab have been demonstrated in patients with NSCLC, RCC, and Hodgkin lymphoma in real-world practice [15–17]. However, to our knowledge, the clinical impact of low-dose nivolumab in patients with advanced HCC is unclear. Therefore, this study aimed to investigate the efficacy and safety of low-dose nivolumab in patients with advanced HCC.

## Materials and methods

### Patients

Data on patients with advanced HCC between January 2019 and December 2021 at the Kaohsiung Chang Gung Memorial Hospital were retrospectively reviewed. The eligibility criteria were as follows: (1) nivolumab 20 or 100 mg Q2W, without combination with other drugs or locoregional therapy such as radiofrequency ablation (RFA), transarterial chemoembolization (TACE), or radiotherapy; (2) no experience with other ICIs (atezolizumab, pembrolizumab, ipilimumab, durvalumab, tremelimumab); (3) no history of previous nivolumab therapy; (4) no history of a second malignancy or concurrent cholangiocarcinoma; (5) exclusion of well-known contraindications to nivolumab, including organ transplantation, autoimmune disease, and human immunodeficiency virus infection; and (6) precise collection of clinical information. Finally, 78 patients with advanced HCC who received low-dose nivolumab therapy were identified.

### Treatment and safety assessment

Patients received nivolumab 20 or 100 mg intravenously Q2W, regardless of body weight, and the choice of dose was determined by what patients could afford. First, we carefully explored the patients’ economic status, and only those who could not afford the cost of standard-dose nivolumab but highly desired this regimen were enrolled. Subsequently, low-dose nivolumab was prescribed after a thorough explanation with full agreement. Treatment was discontinued due to disease progression or occurrence of intolerable adverse events (AEs). During the treatment period, we followed these patients for monitoring and AEs assessment Q2W at the outpatient clinic. Blood tests (including those for evaluating complete blood count, serum biochemistry, thyroid function, and cortisol and glucose levels) and chest radiography were performed regularly. AE and immune-related AE (irAE) grades were assessed according to the National Cancer Institute Common Terminology Criteria for Adverse Events version 5.0 [21].

### Tumor staging and response evaluation

HCC was diagnosed according to pathological findings or the non-invasive criteria of the American Association for the Study of Liver Disease (AASLD) [22, 23]. The Barcelona Clinic Liver Cancer (BCLC) staging classification was used for staging at nivolumab initiation [24]. The albumin-bilirubin (ALBI) score was determined based on serum albumin and total bilirubin levels using the following formula: ALBI score = (log_10_ bilirubin [μmol/L] × 0.66) + (albumin [g/L] × − 0.085). The ALBI score was graded as: grade 1 (≤ − 2.60), grade 2 (− 2.59 to − 1.39), or grade 3 (> − 1.39) [25].

There must be at least one measurable target lesion to evaluate treatment response for each patient with HCC. Dynamic magnetic resonance imaging (MRI) or computed tomography (CT) of the liver was performed every 8–12 weeks after initiation of nivolumab. Treatment response to nivolumab was independently assessed by two radiologists in absence of any medical information in accordance with the Response Evaluation Criteria in Solid Tumors (RECIST) criteria version 1.1 [26].

### Statistical analysis

All data were analyzed using SPSS 22 software (IBM, Armonk, NY, USA). Differences in categorical variables were assessed using the chi-square test. PFS was calculated from the date of nivolumab initiation to the date of disease progression or death from any cause. OS was determined from the date of nivolumab initiation to the date of last visit or death from any cause. The Kaplan–Meier method was used to analyze cumulative survival, and the differences were compared using the log-rank test. Hazard ratios (HRs) with 95% confidence intervals (CIs) were used to estimate prognostic values. All clinicopathological variables with a *p*-values < 0.1 in the univariate analyses were further entered into a multivariate Cox proportional hazards model using a forward stepwise selection to identify independently significant factors. Statistical significance was set at *P* < 0.05.

### Ethics statement

This study was approved by the Institutional Review Board of Chang Gung Medical Foundation (202101199B0) and conducted in accordance with the Declaration of Helsinki. The written informed consent was waived due to the retrospective design of this study.

## Results

### Patient characteristics

Our cohort enrolled 78 patients with advanced HCC who received low-dose nivolumab at Kaohsiung Chang Gung Memorial Hospital between January 2019 and December 2021, including 61 men and 17 women with a median age of 63 years (range: 38–81 years). We documented patient characteristics at nivolumab initiation. All patients were staged as BCLC classification C and had an Eastern Cooperative Oncology Group Performance Status score of 0 or 1. In total, 58 (74.4%) and 20 (25.6%) patients were classified as having Child–Pugh classification A and B (7), respectively. Moreover, 49 (62.8%), 23 (29.5%), and six (7.7%) patients had hepatitis B virus (HBV) infection, hepatitis C virus (HCV) infection, and no viral hepatitis, respectively. The percentages of ALBI 1 and 2 were 42.3 and 57.7%, respectively. The incidence of macrovascular invasion (inferior vena cava, hepatic vein, and portal vein) and extrahepatic spread was 50%, and main portal vein thrombosis was noted in 11 (14.1%) patients. Thirty-one (39.7%) patients underwent hepatectomy before nivolumab treatment, and 27 (34.6%) patients had lymph node metastasis. The median alpha-fetoprotein level was 523 ng/ml (range: < 2 – > 80,000 ng/ml). The baseline patient characteristics are shown in Table 1.

**Table 1:** Characteristics of 78 patients with advanced hepatocellular carcinoma who received low-dose nivolumab

Characteristics	
Age (median, range)	63 (38–81) years
Sex	
Male	61 (78.2%)
Female	17 (21.8%)
ECOG PS	
0	34 (43.6%)
1	44 (56.4%)
Child–Pugh classification	
A	58 (74.4%)
B (7)	20 (25.6%)
BCLC classification	
C	78 (100%)
ALBI grade	
1	33 (42.3%)
2	45 (57.7%)
Viral hepatitis status	
Hepatitis B	49 (62.8%)
Hepatitis C	23 (29.5%)
No	6 (7.7%)
Macrovascular invasion (IVC, HV, PV)	
Yes	39 (50%)
No	39 (50%)
Main portal vein thrombosis	
Yes	11 (14.1%)
No	67 (85.9%)
History of hepatectomy	
Yes	31 (39.7%)
No	47 (60.3%)
Extrahepatic spread	
Yes	39 (50%)
No	39 (50%)
Lymph node metastasis	
Yes	27 (34.6%)
No	51 (65.4%)
AFP (median, range) ng/ml	523 (< 2.0 – > 80,000)

*ECOG PS* Eastern Cooperative Oncology Group Performance Status, *BCLC* Barcelona-Clinic Liver Cancer, *ALBI* Albumin-Bilirubin, *IVC* Inferior vena cava, *HV* Hepatic vein, *PV* Portal vein, *AFP* Alpha-fetoprotein. All status mentioned above were determined at the time of nivolumab initiation

### Response to nivolumab

Treatment response to nivolumab was determined according to the RECIST criteria version 1.1; four (5.1%) patients showed partial response (PR), 21 (26.9%) patients had stable disease (SD), and 53 (68.0%) patients had progressive disease (PD), indicating a disease control rate (DCR) of 32.0%.

There was no statistical difference in the ORR (5.9% vs. 4.9%) and DCR (41.2% vs. 29.5%) between patients treated with nivolumab 20 and 100 mg (*P =* 0.65). Patients who received nivolumab as second-line therapy had a higher ORR (8.9% versus 0%) and DCR (37.8% versus 24.2%), although no statistical significance was noted (*P =* 0.17). The treatment responses to nivolumab are presented in Table 2.

**Table 2:** Treatment response to nivolumab

	All patients (*n =* 78)	Nivolumab 20 mg (*n =* 17)	Nivolumab 100 mg (*n =* 61)	*P* value	Nivolumab second line (*n =* 45)	Nivolumab third and later lines (*n =* 33)	*P* value
Partial response	4 (5.1%)	1 (5.9%)	3 (4.9%)	0.65	4 (8.9%)	0 (0%)	0.17
Stable disease	21 (26.9%)	6 (35.3%)	15 (24.6%)		13 (28.9%)	8 (24.2%)	
Progressive disease	53 (68.0%)	10 (58.8%)	43 (70.5%)		28 (62.2%)	25 (75.8%)	
Disease control rate	25 (32.0%)	7 (41.2%)	18 (29.5%)		17 (37.8%)	8 (24.2%)	

### Clinical outcomes

**Fig. 1:**
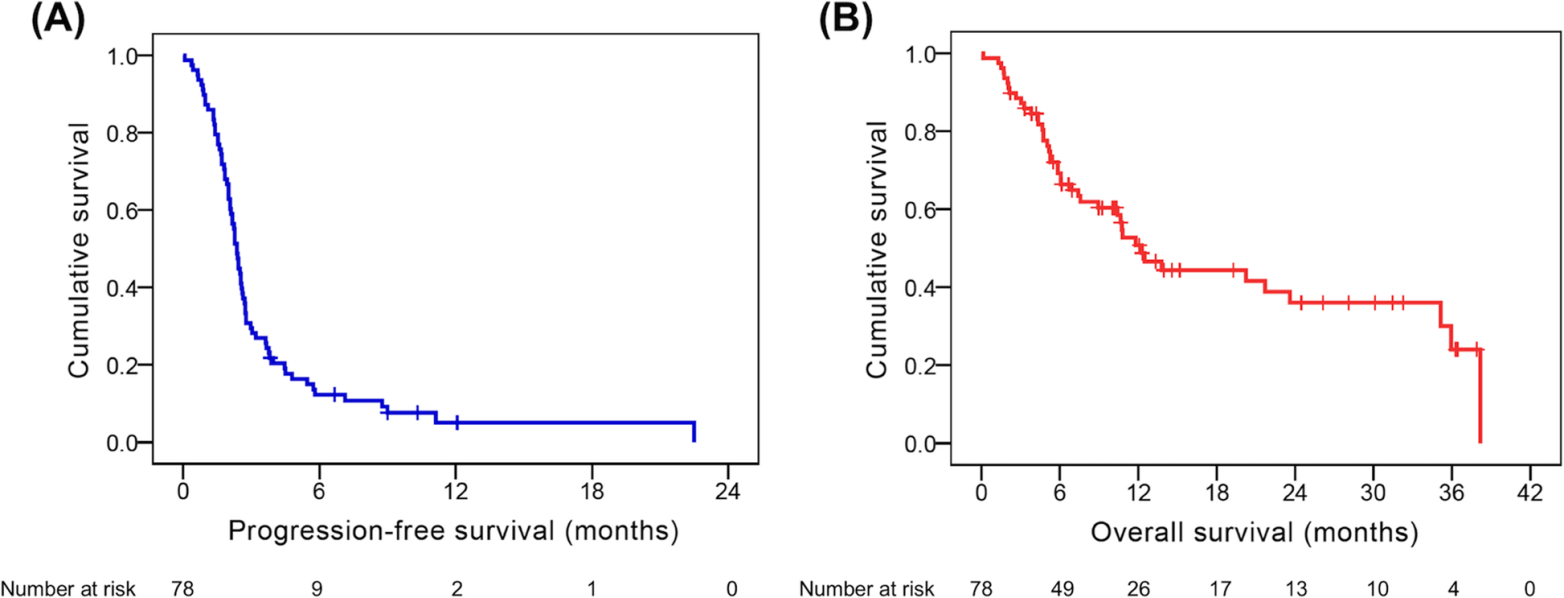
Kaplan–Meier curves for progression-free survival (PFS) and overall survival (OS) in patients with advanced hepatocellular carcinoma who received low-dose nivolumab. **A** PFS and (**B**) OS

The baseline clinicopathologic factors did not differ significantly between nivolumab 20 mg and nivolumab 100 mg groups except ECOG PS; patients who received nivolumab 100 mg were mentioned to have higher percentage of ECOG PS 0 compared to those with nivolumab 20 mg (Table 3). The median PFS and OS were 2.4 and 12.3 months, respectively (Fig. 1).

**Table 3:** Comparison of clinicopathological parameters in 78 patients with advanced hepatocellular carcinoma who received low-dose nivolumab

Characteristics	Nivolumab 100 mg (n = 61)	Nivolumab 20 mg (n = 17)	*P* value
Age			0.19
< 60 years	25 (41.0%)	4 (23.5%)	
≥ 60 years	36 (59.0%)	13 (76.5%)	
Sex			0.26
Male	15 (24.6%)	2 (11.8%)	
Female	46 (75.4%)	15 (88.2%)	
ECOG PS			0.015*
0	31 (50.8%)	3 (17.6%)	
1	30 (49.2%)	14 (82.4%)	
Child–Pugh classification			0.30
A	47 (77.0%)	11 (64.7%)	
B (7)	14 (23.0%)	6 (35.3%)	
ALBI grade			0.51
1	27 (44.3%)	6 (35.3%)	
2	34 (55.7%)	11 (64.7%)	
Hepatitis B			0.13
Yes	41 (67.2%)	8 (47.1%)	
No	20 (32.8%)	9 (52.9%)	
Hepatitis C			0.07
Yes	15 (24.6%)	8 (47.1%)	
No	46 (75.4%)	9 (52.9%)	
Macrovascular invasion (IVC, HV, PV)			0.41
Yes	29 (47.5%)	10 (58.8%)	
No	32 (52.5%)	7 (41.2%)	
Main portal vein thrombosis			0.75
Yes	9 (14.8%)	2 (11.8%)	
No	52 (85.2%)	15 (88.2%)	
History of hepatectomy			0.89
Yes	24 (39.3%)	7 (41.2%)	
No	37 (60.7%)	10 (58.8%)	
Extrahepatic spread			0.41
Yes	32 (52.5%)	7 (41.2%)	
No	29 (47.5%)	10 (58.8%)	
Lymph node metastasis			0.95
Yes	21 (34.4%)	6 (35.3%)	
No	40 (65.6%)	11 (64.7%)	
Treatment lines			0.32
Second line	37 (60.7%)	8 (47.1%)	
Third line and later lines	24 (39.3%)	9 (52.9%)	
AFP ≥ 400 ng/ml			0.29
Yes	34 (55.7%)	7 (41.2%)	
No	27 (44.3%)	10 (58.8%)	

*ECOG PS* Eastern Cooperative Oncology Group Performance Status, *BCLC* Barcelona-Clinic Liver Cancer, *ALBI* Albumin-Bilirubin, *IVC* Inferior vena cava, *HV* Hepatic vein, *PV* Portal vein, *AFP* Alpha-fetoprotein. All status mentioned above were determined at the time of nivolumab initiation. *Statistical difference

**Fig. 2:**
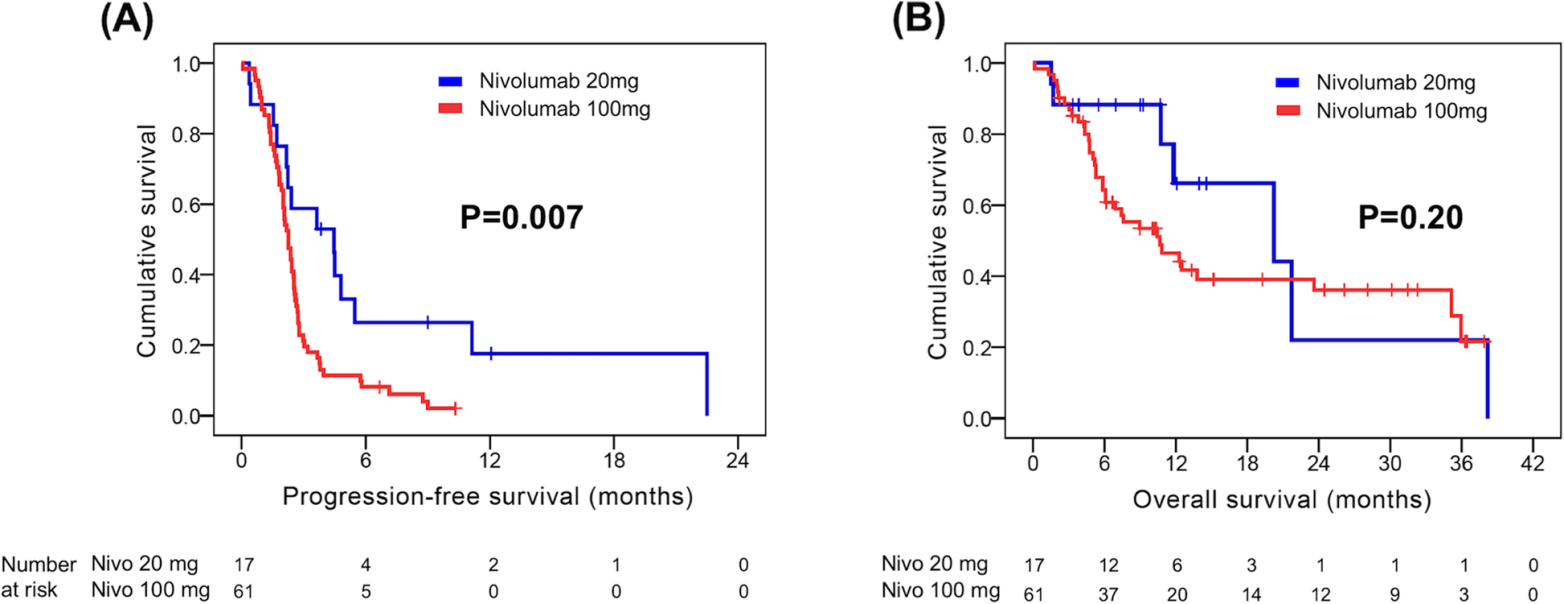
Comparison of progression-free survival (PFS) and overall survival (OS) by nivolumab dose. **A** PFS and (**B**) OS

**Fig. 3:**
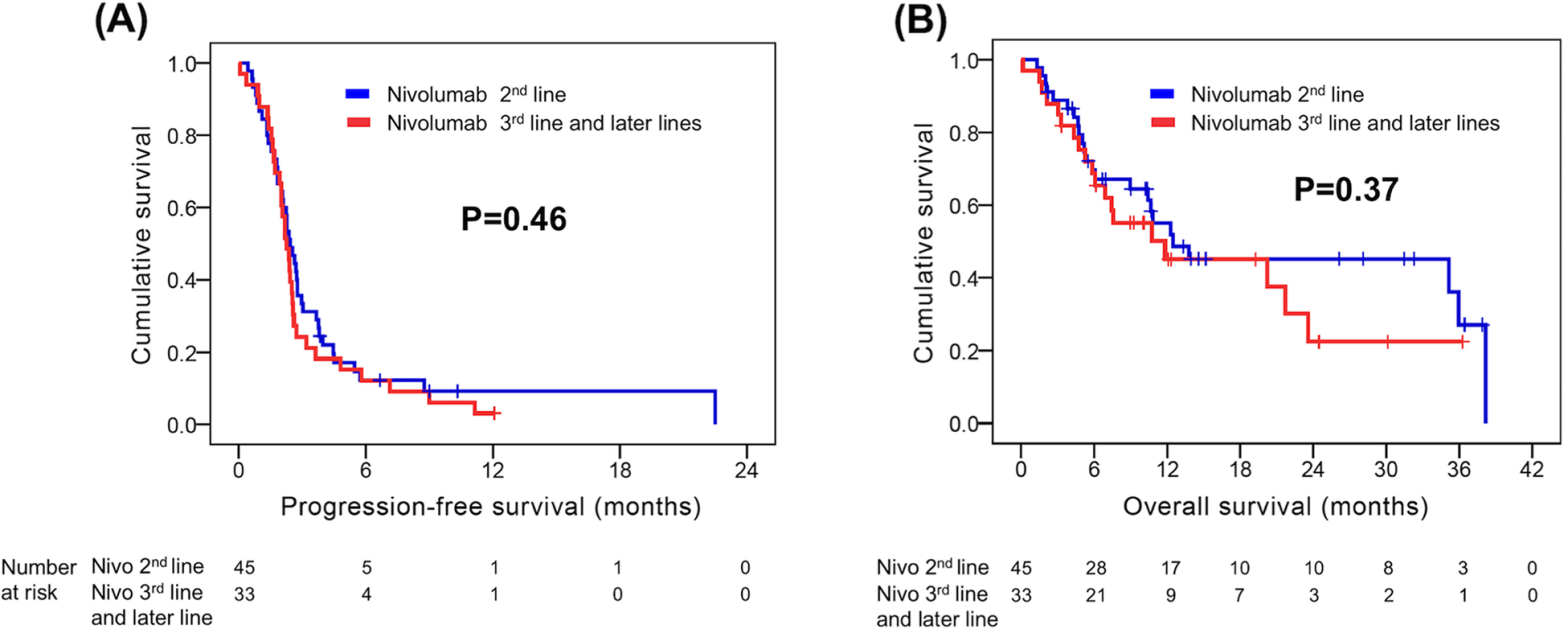
Kaplan–Meier curves for progression-free survival (PFS) and overall survival (OS) according to nivolumab treatment lines. **A** PFS and (**B**) OS

Regarding PFS, no statistical significance was observed in any parameters in univariate analysis, except nivolumab dose. The 17 patients who received nivolumab 20 mg had a superior PFS than those who received nivolumab 100 mg (4.5 months versus 2.3 months, *P =* 0.007, Fig. 2A). The median PFS was comparable between patients treated with nivolumab as second-line treatment and those treated with nivolumab as third-line and later-line lines (2.4 months versus 2.3 months, Fig. 3A) Multivariate analysis also revealed that nivolumab 20 mg (*P =* 0.009; HR, 0.43; 95% CI, 0.22–0.81) was an independent prognostic factor for better PFS. Univariate and multivariate analysis results for PFS are presented in Table 4.

**Table 4:** Univariate and multivariate analysis results for PFS in 78 patients with advanced hepatocellular carcinoma who received low-dose nivolumab

Characteristics	No. of patients	Univariate		Multivariate	
		PFS (months)	*P* value	HR (95% CI)	*P* value
Age					
< 60 years	29 (37.2%)	2.3	0.68		
≥ 60 years	49 (62.8%)	2.4			
Sex					
Male	61 (78.2%)	2.4	0.99		
Female	17 (21.8%)	2.4			
ECOG PS					
0	34 (43.6%)	2.3	0.11		
1	44 (56.4%)	2.4			
Child–Pugh classification					
A	58 (74.4%)	2.3	0.29		
B (7)	20 (25.6%)	2.4			
ALBI grade					
1	33 (42.3%)	2.2	0.56		
2	45 (57.7%)	2.4			
Hepatitis B					
Yes	49 (62.8%)	2.4	0.27		
No	29 (37.2%)	2.4			
Hepatitis C					
Yes	23 (29.5%)	2.3	0.48		
No	55 (70.5%)	2.4			
Macrovascular invasion (IVC, HV, PV)					
Yes	39 (50%)	2.4	0.43		
No	39 (50%)	2.4			
Main portal vein thrombosis					
Yes	11 (14.1%)	2.2	0.09		
No	67 (85.9%)	2.4			
History of hepatectomy					
Yes	31 (39.7%)	2.5	0.13		
No	47 (60.3%)	2.3			
Extrahepatic spread					
Yes	39 (50%)	2.0	0.06		
No	39 (50%)	2.6			
Lymph node metastasis					
Yes	27 (34.6%)	2.5	0.51		
No	51 (65.4%)	2.2			
Nivolumab dose					
20 mg	17 (21.8%)	4.5	0.007*	0.43 (0.22–0.81)	0.009*
100 mg	61 (78.2%)	2.3			
Treatment lines					
Second line	45 (57.7%)	2.4	0.46		
Third line and later lines	33 (42.3%)	2.3			
AFP ≥ 400 ng/ml					
Yes	41 (52.6%)	2.4	0.41		
No	37 (47.4%)	2.3			

*PFS* Progression-free survival, *ECOG PS* Eastern Cooperative Oncology Group Performance Status, *BCLC* Barcelona-Clinic Liver Cancer, *ALBI* Albumin-Bilirubin, *IVC* Inferior vena cava, *HV* Hepatic vein, *PV* Portal vein, *AFP* Alpha-fetoprotein. All status mentioned above were determined at the time of nivolumab initiation. *Statistical difference

In univariate analysis for OS, there were no significant differences in any parameters, except Child–Pugh classification and ALBI grade. Superior OS was noted in patients with Child–Pugh classification A than in those with Child–Pugh classification B (7) (20.2 months versus 5.2 months, *P =* 0.022). The 33 patients with ALBI grade 1 had better OS than the 45 patients with ALBI grade 2 (35.9 months versus 10.8 months, *P =* 0.017). In addition, there was no significant difference in OS between the different nivolumab dose (Fig. 2B) or different treatment lines (Fig. 3B). ALBI grade 1 (*P =* 0.020; HR, 0.46; 95% CI, 0.24–0.88) was the only independent prognostic factor for superior OS in multivariate analysis. Univariate and multivariate analysis results for OS are shown in Table 5.

**Table 5:** Univariate and multivariate analyses of OS in 78 patients with advanced hepatocellular carcinoma who received low-dose nivolumab

Characteristics	No. of patients	Univariate		Multivariate	
		OS (months)	*P* value	HR (95% CI)	*P* value
Age					
< 60 years	29 (37.2%)	7.6	0.26		
≥ 60 years	49 (62.8%)	20.2			
Sex					
Male	61 (78.2%)	12.3	0.82		
Female	17 (21.8%)	20.2			
ECOG PS					
0	34 (43.6%)	12.3	0.36		
1	44 (56.4%)	11.8			
Child–Pugh classification					
A	58 (74.4%)	20.2	0.022*		
B (7)	20 (25.6%)	5.2			
ALBI grade					
1	33 (42.3%)	35.9	0.017*	0.46 (0.24–0.88)	0.020*
2	45 (57.7%)	10.8			
Hepatitis B					
Yes	49 (62.8%)	10.8	0.60		
No	29 (37.2%)	20.2			
Hepatitis C					
Yes	23 (29.5%)	11.8	0.95		
No	55 (70.5%)	12.3			
Macrovascular invasion (IVC, HV, PV)					
Yes	39 (50%)	20.2	0.88		
No	39 (50%)	10.8			
Main portal vein thrombosis					
Yes	11 (14.1%)	6.1	0.09		
No	67 (85.9%)	13.8			
History of hepatectomy					
Yes	31 (39.7%)	38.2	0.07		
No	47 (60.3%)	10.8			
Extrahepatic spread					
Yes	39 (50%)	20.2	0.50		
No	39 (50%)	10.8			
Lymph node metastasis					
Yes	27 (34.6%)	20.2	0.49		
No	51 (65.4%)	10.8			
Nivolumab dose					
20 mg	17 (21.8%)	20.2	0.20		
100 mg	61 (78.2%)	10.6			
Treatment lines					
Second line	45 (57.7%)	12.5	0.37		
Third line and later lines	33 (42.3%)	11.8			
AFP ≥400 ng/ml					
Yes	41 (52.6%)	10.7	0.34		
No	37 (47.4%)	21.7			

*OS* Overall survival, *ECOG PS* Eastern Cooperative Oncology Group Performance Status, *BCLC* Barcelona-Clinic Liver Cancer, *ALBI* Albumin-Bilirubin, *IVC* Inferior vena cava, *HV* Hepatic vein, *PV* Portal vein, *AFP* Alpha-fetoprotein. All status mentioned above were determined at the time of nivolumab initiation. *Statistical difference

### Efficacy based on HBV and HCV

**Fig. 4:**
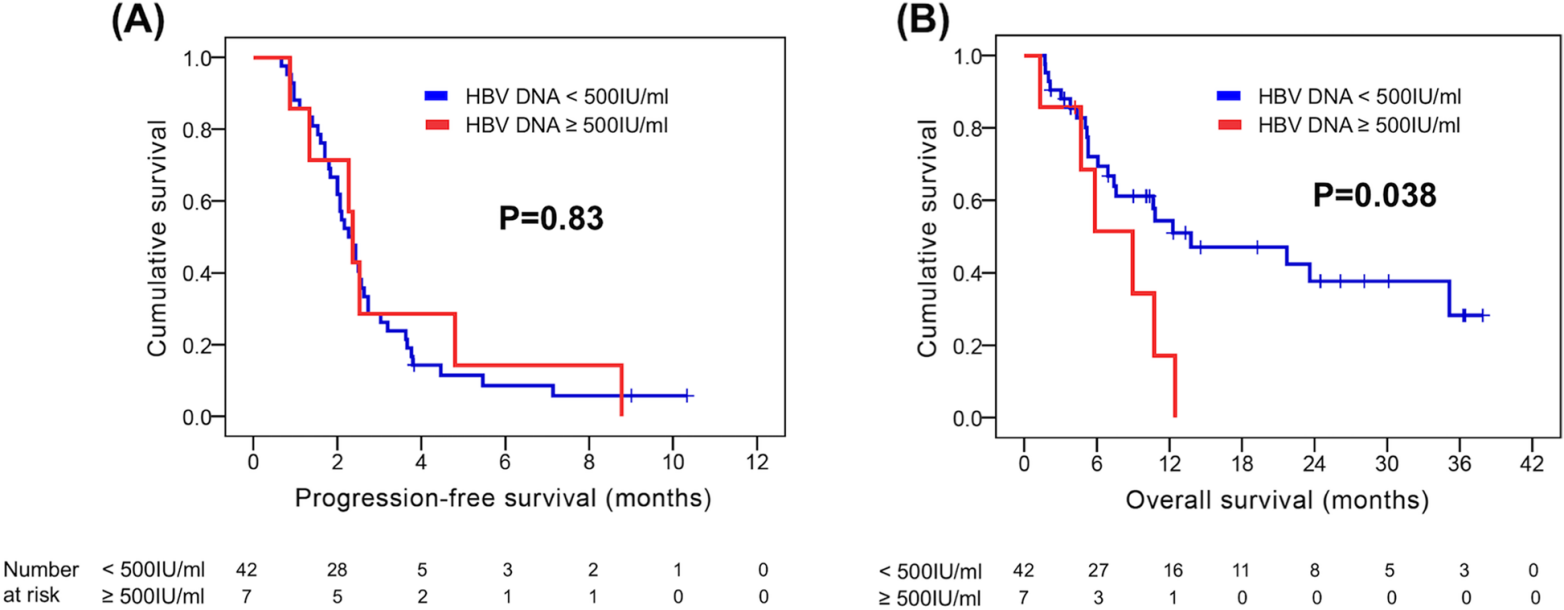
Comparison of progression-free survival (PFS) and overall survival (OS) according to the viral hepatitis B DNA titer. **A** PFS and (**B**) OS

The 49 patients with HBV were divided into two groups according to their HBV viral load status: HBV DNA ≥500 IU/ml and HBV DNA < 500 IU/ml. The median PFS was comparable between patients with HBV DNA ≥500 IU/ml and those with HBV DNA < 500 IU/ml (Fig. 4A). However, patients with HBV DNA < 500 IU/ml had superior OS than those with HBV DNA ≥500 IU/ml (13.8 months versus 8.9 months, *P =* 0.038, Fig. 4).

**Fig. 5:**
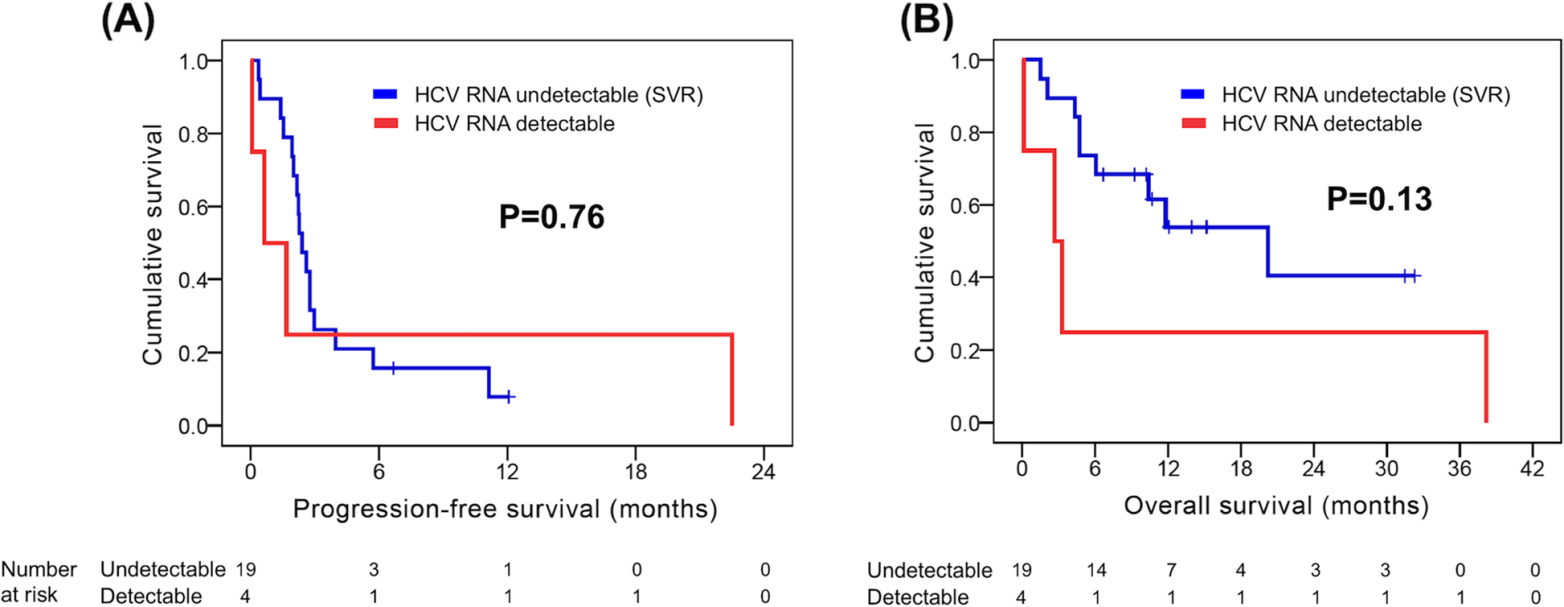
Comparison of progression-free survival (PFS) and overall survival (OS) according to viral hepatitis C RNA presentation. **A** PFS and (**B**) OS. SVR: sustained virological response

Among then 23 patients with HCV, 19 patients showed sustained virological response (SVR) and four patients had detectable HCV RNA (non-SVR or treatment-naïve). Patients with SVR had a longer PFS (2.4 months versus 0.6 months) and OS (20.2 months versus 2.6 months) than those with detectable HCV RNA, although no statistical difference was noted (Fig. 5).

### Safety assessment

The most common AEs were fatigue (11.5%), rash (8.9%), pruritus (6.4%), diarrhea (5.1%), increased aspartate/alanine aminotransferase levels (5.1%), decreased appetite (3.8%), decreased body weight (2.6%), nausea (2.6%), hypothyroidism (2.6%), hypersensitivity/infusion-related reaction (2.6%), and hyperthyroidism (1.3%). All AEs were grade 1–2 in severity; there were no grade 3–4 toxicities or drug-related grade 5 AEs. Immune-modulating therapy and systemic corticosteroids were not required. All patients tolerated the AEs of nivolumab without treatment interruption or dose adjustment due to AEs. The median onset of irAE showed that skin toxicity was 36 days (range: 14-71 days), colitis was 47 days (range: 28-62 days), liver toxicity was 63 days (range: 28-84 days) and endocrinopathy was 105 days (range: 70-140 days). The timing and frequency of irAE in our study was similar with previous reports [13, 27]. The incidence of drug-related AEs is presented in Table 6.

**Table 6:** Incidence of treatment-related adverse events in 78 patients with advanced hepatocellular carcinoma who underwent low-dose nivolumab

Adverse event	Any grades	Grade 3/4
Rash	7 (8.9%)	0 (0%)
Pruritis	5 (6.4%)	0 (0%)
Diarrhea	4 (5.1%)	0 (0%)
Decreased appetite	3 (3.8%)	0 (0%)
Fatigue	9 (11.5%)	0 (0%)
Decreased body weight	2 (2.6%)	0 (0%)
Aspartate/Alanine aminotransferase increase	4 (5.1%)	0 (0%)
Nausea	2 (2.6%)	0 (0%)
Hyperthyroidism	1 (1.3%)	0 (0%)
Hypothyroidism	2 (2.6%)	0 (0%)
Pneumonitis	0 (0%)	0 (0%)
Hypersensitivity/infusion-related reaction	2 (2.6%)	0 (0%)

### Patient disposition

Thirty-one (39.7%) patients received nivolumab treatment. Regarding targeted therapy, lenvatinib, sorafenib, regorafenib, ramucirumab, and thalidomide were administered to seven (9.0%), three (3.8%), three (3.8%), five (6.4%), and one (1.3%) patients, respectively. Chemotherapy included the FOLFOX regimen, epirubicin, and gemcitabine in 10 (12.8%), three (3.8%), and two (2.6%) patients, respectively. Four (5.1%) patients received ICIs (atezolizumab plus bevacizumab) after nivolumab treatment. The profiles of post-nivolumab treatments are presented in Table 7.

**Table 7:** Subsequent therapy after nivolumab progression

Category	(*n =* 78)
Any post-nivolumab anti-cancer treatment	31 (39.7%)
Targeted therapy	
Lenvatinib	7 (9.0%)
Sorafenib	3 (3.8%)
Regorafenib	3 (3.8%)
Ramucirumab	5 (6.4%)
Thalidomide	1 (1.3%)
Chemotherapy	
FOLFOX	10 (12.8%)
Epirubicin	3 (3.8%)
Gemcitabine	2 (2.6%)
Immune checkpoint inhibitors	
Atezolizumab plus bevacizumab	4 (5.1%)

## Discussion

Our study demonstrated the efficacy and safety of low-dose nivolumab (20 or 100 mg) in a real-world setting for the management of advanced HCC. The ORR, DCR, median PFS, and median OS were 5.1, 32%, 2.4 months, and 12.3 months, respectively. There was no statistical difference in OS in the setting of nivolumab dose or nivolumab treatment lines, although a poorer ORR was found in patients treated with nivolumab as a third- and later-line treatment than that in patients who received nivolumab as a second-line treatment. Additionally, the safety profile revealed good tolerability without grade 3–4 toxicities. Further, superior OS was observed in patients with better HBV or HCV control. Based on the assumption that lower doses result in lower costs, the findings of our study suggest that low-dose nivolumab is both economically and clinically worth considering as a treatment option for patients with HCC struggling to afford the standard dose of nivolumab.

There is a lack of evidence to prove the dose–response relationship or a maximum tolerated dose (MTD) in numerous early phase studies of nivolumab or pembrolizumab. In the CheckMate 040 study, a dose-escalation and expansion study, nivolumab was administered at a dose of 0.1 mg/kg 10 mg/kg Q2W for HCC treatment, showing a manageable safety profile and tolerability, but no MTD was confirmed [11]. Subsequently, the FDA approved nivolumab at a fixed dose of 240 mg Q2W for all approved indications regardless of body weight. However, the extremely high cost of the standard-dose nivolumab, also termed financial toxicity, limited the use of nivolumab in patients with HCC who could not afford it. In contrast, another study confirmed similar efficacy and safety between a flat dose of 240 mg nivolumab and a dose of 3 mg/kg nivolumab based on pharmacokinetic and dose efficacy analyses, indicating that a lower flat dose nivolumab (20 or 100 mg) might be comparably effective to a higher dose [28]. Therefore, growing evidence has confirmed the efficacy and safety of low-dose ICIs to reduce the financial toxicity and improve clinical outcome in several cancer types, such as NSCLC, RCC, and Hodgkin lymphoma [15–17]. In NSCLC, Yoo et al. reported 47 patient who received low-dose nivolumab (20 or 100 mg) or standard dose (3 mg/kg) for cancer treatment; there was no statistical difference of ORR (13.8% versus 16.7%), PFS (3.0 month versus 1.0 month) and OS (12.5 month versus 8.2 month) between low-dose group and standard dose groups [16]. In another study which focused on the RCC, no difference in ORR (50.0% versus 43.8%), PFS (7.0 month versus 7.0 month) and OS (not reached versus 28.0 month) was observed in patients with low-dose (1.7 mg/kg) nivolumab and high-dose (2.7 mg/kg) nivolumab [17]. In these two studies, the nivolumab dosing is around 100 mg (even 20 mg in the NSCLC study), similar with the dose of nivolumab in our HCC cohort; in addition, there was no significant difference in the analyses of ORR, PFS and OS, suggesting the potential benefit of low-dose nivolumab in clinical practice. Our study also demonstrated the clinical benefit and safety profile of low-dose nivolumab in real-world practice of HCC management.

The median OS in our study is relatively short compared to that in the CheckMate 040 trial (12.3 months versus 15.1 months) [11]. The reasons may be as follows. First, there were near 25% patients with Child–Pugh classification B (7) in our study, but almost all patients enrolled in the CheckMate 040 trials were classified as having Child–Pugh classification A. Second, subsequent therapy after nivolumab progression in our study was relatively lower than that in the CheckMate 040 study (39.7% versus 52%). In our study, low-dose nivolumab was used because the patients could not afford the costs of standard-dose nivolumab. Therefore, in this situation, subsequent therapy after nivolumab progression would also be unaffordable for them. Third, vascular invasion is a well-known poor prognostic factor in HCC; the incidence of macrovascular invasion was high (50%) in our study, but that in the CheckMate 040 trial was 30%, which might have resulted in a worse OS. Fourth, the CheckMate 040 study reported that 71% of the whole population had extrahepatic metastasis, but the percentage of extrahepatic spread was only 50%. Hepatic lesions may be less responsive to ICIs than extrahepatic lesions [29].

The ORR was only 5.1% in our study, while that in the CheckMate 040 trial was 13% [11, 20]. Regarding the lower ORR in our study, some situations may explain this result. First, we enrolled nearly 25% of the entire population with Child–Pugh classification B (7). In the CheckMate 040 cohort 5, a phase I/II study of nivolumab in patients with Child–Pugh B cirrhosis, the ORR was 12%, which was slightly lower than that in the CheckMate 040 study [30]. Therefore, poor liver preservation (Child-–Pugh B) may result in a worse ORR. Second, as mentioned above, hepatic lesions may be less responsive to ICIs than extrahepatic lesions [29]. Only 50% patients had extrahepatic spread in our study, but 71% patients had extrahepatic metastasis in the CheckMate 040 study; this might be another reason for the poor ORR data in our study.

HBV infection is a predominant risk factor for HCC in some Asian countries, and antiviral therapy to suppress HBV has been proven to improve survival and reduce recurrence in patients with HCC undergoing surgical resection, TACE, RFA, or liver transplantation [31–34]. In our study, the median PFS was approximately 2.4 months regardless of HBV DNA titer; however, superior OS was found in patients with HBV DNA < 500 IU/ml than in those with HBV DNA ≥500 IU/ml. However, growing evidence has demonstrated that the successful treatment of HCC is associated with a marked improvement in patients with HCC [35]. In addition, another study that retrospectively reviewed 22,500 patients with HCV showed that SVR might contribute to a risk reduction of 76% for the development of HCC compared to non-SVR [36]. Our study also revealed similar results, showing superior PFS and OS in patients with SVR than in those without SVR or treatment-naïve patients, although there was no statistical difference.

Our study has several limitations. First, it was a retrospective analysis with a relatively small sample size of patients, which might have resulted in low statistical power. Second, the duration of the follow-up period might not have been long enough, contributing to the difficulty in concluding a statistically significant difference in risk factors. Third, there was a lack of patients who received a standard dose of nivolumab (240 or 3 mg/kg) as a control. Fourth, the number of patients treated with low-dose nivolumab depends on the economic status, which means that the patient could not pay the high cost of the standard dose of nivolumab, which might be a potential bias. However, to our knowledge, this is one of the few studies that investigated the efficacy and safety of low-dose ICI in the management of HCC in real-world practice. The clinical benefit of ICIs in the management of HCC has been approval in many phase III randomized controlled trials; however, the high medical cost is a persistent issue because most patients could not afford the standard dose of ICIs in Taiwan. In order to improve survival benefit from the treatment of ICIs in HCC patients, it is critical to promote the reimbursement of ICIs for HCC by national health insurance system in Taiwan. On the other hand, in order to more understand the efficacy and toxicity between low-dose and standard dose ICIs, a multi-center data collection with propensity score matching method to decrease selection bias may be feasible and helpful.

## Conclusions

Low-dose nivolumab may be effective with manageable toxicity and can be an alternative option to reduce financial toxicity in patients with advanced HCC who cannot afford the high cost of ICIs in real-world practice. Further larger prospective studies with sufficient sample sizes are warranted to validate our results regarding low-dose nivolumab therapy.

## Data Availability

The datasets used and analyzed during the current study are available from the corresponding author on reasonable request.

## Abbreviations

HCC: Hepatocellular carcinoma
ICI: Immune checkpoint inhibitor
PD-1: Programmed death 1
NSCLC: Non-small cell lung cancer
ORR: Objective response rate
PFS: Progression-free survival
OS: Overall survival
FDA: Food and Drug Administration
RCC: Recall cell carcinoma
Q2W: Every 2 weeks
RFA: Radiofrequency ablation
TACE: Transarterial chemoembolization
AE: Adverse event
irAE: Immune-related adverse event
AASLD: American Association for the Study of Liver Disease
BCLC: Barcelona Clinic Liver Cancer
ALBI: Albumin-bilirubin
CT: Computed tomography
MRI: Magnetic resonance imaging
RECIST: Response Evaluation Criteria in Solid Tumors
HR: Hazard ratio
CI: Confidence interval
HBV: Hepatitis B virus
HCV: Hepatitis C virus
PR: Partial response
SD: Stable disease
PD: Progressive disease
DCR: Disease control rate
SVR: Sustained virological response
MTD: Maximum tolerated dose
